# Duration of Life among Medical Men

**Published:** 1853

**Authors:** 


*Duration of Life among Medical Men.*

According to Dr. Guy, the duration of life is greater among physicians and surgeons than among the general practitioners of medicine and surgery. 2. That this great longevity of the former is only in part explained by a less amount of exposure to contagious diseases and other professional risks. 3. That the duration of life of members of the medical profession (being chiefly physicians and surgeons) does not differ materially from the duration of life of the clergy. 4. That the duration of life of medical men has somewhat increased during the last three centuries.

Athenæum, January, 1854.

